# Single cell RNA sequencing of human FAPs reveals different functional stages in Duchenne muscular dystrophy

**DOI:** 10.3389/fcell.2024.1399319

**Published:** 2024-07-09

**Authors:** Esther Fernández-Simón, Patricia Piñol-Jurado, Rasya Gokul-Nath, Adrienne Unsworth, Jorge Alonso-Pérez, Marianela Schiava, Andres Nascimento, Giorgio Tasca, Rachel Queen, Dan Cox, Xavier Suarez-Calvet, Jordi Díaz-Manera

**Affiliations:** ^1^ John Walton Muscular Dystrophy Research Centre, Newcastle University Translational and Clinical Research Institute and Newcastle Hospitals NHS Foundation Trust, NE1 3BZ, Newcastle Upon Tyne, United Kingdom; ^2^ Bioinformatics Unit, Newcastle University, Newcastle Upon Tyne, United Kingdom; ^3^ Neuromuscular Disorders Unit, Neurology Department, Hospital Sant Joan de Deu, Barcelona, Spain; ^4^ Centro de Investigación Biomédica en Red en Enfermedades Raras (CIBERER), Madrid, Spain; ^5^ Neuromuscular Disorders Unit, Neurology Department, Insitut de Recerca de l’Hospital de la Santa Creu I Sant Pau, Barcelona, Spain; ^6^ Neuromuscular Disease Unit, Neurology Department, Hospital Universitario Nuestra Señora de Candelaria, Fundación Canaria Instituto de Investigación Sanitaria de Canarias (FIISC), Tenerife, Spain

**Keywords:** muscle dystrophies, fibro-adipogenic progenitor cells, fibrosis, adipogenesis, single cell RNA sequencing

## Abstract

**Background:** Duchenne muscular dystrophy is a genetic disease produced by mutations in the dystrophin gene characterized by early onset muscle weakness leading to severe and irreversible disability. Muscle degeneration involves a complex interplay between multiple cell lineages spatially located within areas of damage, termed the degenerative niche, including inflammatory cells, satellite cells (SCs) and fibro-adipogenic precursor cells (FAPs). FAPs are mesenchymal stem cell which have a pivotal role in muscle homeostasis as they can either promote muscle regeneration or contribute to muscle degeneration by expanding fibrotic and fatty tissue. Although it has been described that FAPs could have a different behavior in DMD patients than in healthy controls, the molecular pathways regulating their function as well as their gene expression profile are unknown.

**Methods:** We used single-cell RNA sequencing (scRNAseq) with 10X Genomics and Illumina technology to elucidate the differences in the transcriptional profile of isolated FAPs from healthy and DMD patients.

**Results:** Gene signatures in FAPs from both groups revealed transcriptional differences. Seurat analysis categorized cell clusters as proliferative FAPs, regulatory FAPs, inflammatory FAPs, and myofibroblasts. Differentially expressed genes (DEGs) between healthy and DMD FAPs included upregulated genes CHI3L1, EFEMP1, MFAP5, and TGFBR2 in DMD. Functional analysis highlighted distinctions in system development, wound healing, and cytoskeletal organization in control FAPs, while extracellular organization, degradation, and collagen degradation were upregulated in DMD FAPs. Validation of DEGs in additional samples (*n* = 9) using qPCR reinforced the specific impact of pathological settings on FAP heterogeneity, reflecting their distinct contribution to fibro or fatty degeneration *in vivo*.

**Conclusion:** Using the single-cell RNA seq from human samples provide new opportunities to study cellular coordination to further understand the regulation of muscle homeostasis and degeneration that occurs in muscular dystrophies.

## 1 Introduction

Duchenne muscular dystrophy (DMD) is a genetic disease produced by variants in the *DMD* gene leading to a reduced expression or absence of the subsarcolemmal protein, dystrophin. DMD is characterized by early onset muscle weakness progressing towards loss of ambulation between the ages of 8–14 years old and is associated to cardiac and respiratory dysfunction which, ultimately, lead to premature death (Birnkrant et al., 2018; Mercuri et al., 2019).

Lack of dystrophin makes the muscle fibers instable and susceptible to damage during muscle contraction (Mahdy, 2018; Biferali et al., 2019). Muscle fibre damage triggers a cascade of molecular consequences including the activation of proteases, increase in reactive nitrogen species and release of proinflammatory cytokines to the stroma that contribute to the infiltration of muscle by inflammatory cells (Del Rocío Cruz-Guzmán et al., 2015). Macrophages are the main inflammatory cell infiltrating the muscle and interacting with muscle resident cells including satellite cells (SCs) and also fibro-adipogenic progenitor cells (FAPs) (De Paepe and De Bleecker, 2013; Contreras et al., 2016). Activated SCs proliferate and differentiate, fusing with damaged muscle fibres and contributing to their regeneration. Conversely, FAPs proliferate and differentiate into fibrotic or adipogenic cells leading to expansion of fibrotic and fat tissue that substitutes lost muscle fibres contributing to the degeneration of skeletal muscle observed in muscular dystrophies (Uezumi et al., 2014; Ieronimakis et al., 2016; Malecova et al., 2018). The expansion of the fibrofatty tissue in the skeletal muscle of patients with DMD has a series of negative consequences including its contribution to the development of permanent muscle weakness and contractures, impairment of the capacity of SCs to proliferate and differentiate effectively and, reducing the response of muscles to new therapies.

FAPs have been a matter of research in recent years as they seem to be pivotal in the regeneration and degeneration processes in neuromuscular diseases. FAPs are muscle resident mesenchymal stem cells characterized by the expression of platelet derived growth factor receptor alpha (PDGFRα). FAPs are activated after muscle damage, proliferate and then differentiate into several types of cells including pro-regenerative FAPs, fibroblast and adipocytes (Arrighi et al., 2015; Marcelin et al., 2017). Although it has been described that FAPs could have a different behaviour in DMD patients than in healthy controls, the molecular pathways regulating their function as well as their gene expression profile are unknown. Moreover, it is not known if there are subtypes of FAPs or at least FAPs at different stages of cell differentiation that could be involved in diverse biological processes coexisting in the skeletal muscles of patients with DMD. In recent years there has been a considerable progress in the understanding of how FAPs are activated and differentiate thanks to the studies performed in murine models of acute muscle damage or muscular dystrophies (Zhou et al., 2006; Huang et al., 2009). However, the results observed in murine models have not been validated in human samples.

Single cell and single nuclei RNA sequencing are new transcriptomic technologies that allow the analysis of the gene expression profile in individual cells from tissues or from cells in culture (Rubenstein et al., 2020). These are powerful tools that can be used to identify new cell types based in the expression of specific genes but also facilitate the recognition of molecular pathways that are dysregulated in disease condition helping to understand how the disease process is orchestrated and what are the consequences of specific misfunctions in different tissues.

In this study, we used single cell RNA sequencing (scRNAseq) to study FAPs isolated from muscle biopsies of patients with DMD and healthy controls to elucidate the differences in their transcriptional profile with the aim to better understand whether there are FAPs at different stages of differentiation, the differences in their gene expression profile and their potential role in the process of muscle degeneration.

## 2 Methods

### 2.1 Patients and muscle samples

Muscle biopsies were obtained from three boys with a genetic diagnosis of DMD and two healthy boys. Clinical data of the patients are described in Table 1.

**TABLE 1:** Clinical and genetic data of DMD patients and control included in the study.

Patient	Age at muscle biopsy	Biopsy site	Variant in the *DMD* gene	Ambulatory status at the time of muscle biopsy	Glucocorticoid status
Healthy control 1	12	Quadriceps	Not applicable	Ambulant	No
Healthy control 2	10	Gracilis	Not applicable	Ambulant	No
DMD 1	10	Biceps brachii	Deletion 49–52	Ambulant	Yes
DMD 2	9	Biceps brachii	Deletion 18–44	Ambulant	Yes
DMD 3	9	Biceps brachii	Deletion 45–52	Ambulant	Yes

Inform consent form parents/legal guardians and boys’ assent forms were obtained for donating these samples to research. Muscle samples from the biceps brachii of age and gender matched controls were provided by the orthopaedic surgery department of Hospital de la Santa Creu I Sant Pau in Barcelona. The study was approved by the Ethics Committee of the Hospital de la Santa Creu I Sant Pau Hospital.

### 2.2 Cell culture

Frozen muscle explants from healthy controls and DMD patients were cultured to obtain myoblasts and FAPs as previously described (Suárez-Calvet et al., 2021). Briefly, muscle fragments were cultured in gelatin-plasma coated dishes with DMEM-GlutaMAX (ThermoFisher [Gibco], Waltham, MA) supplemented with 20% FBS (Gibco), 1%PS (Lonza, Basilea, Switzerland)) and 2.5 ng/mL of basic fibroblast growth factor (Peprotech, Rocky Hill, NJ). Sprouting cells from explants were trypsinized (Gibco), expanded and subcultured in Cell + culture flasks (Sarstedt, Nümbrecht, Germany) for 6 days.

### 2.3 Cell sorting

Cultured human muscle derived cells were labelled using anti-PDGFRα (BAF322, R&D Systems, Minneapolis, MN) followed by streptavidine-PECy5 (405202, Biolegend, San Diego, CA) and anti-CD56 (Milteny-Biotech). Samples were acquired with the MACSQuant Analyzer 10 flow cytometer (MiltenyiBiotec). Stained cells were sorted on a FACSAria using FACSDiva software (Becton Dickinson, Ashland, OR). Doublet cells were excluded using forward scatter area and height and fluorescence minus one (FMO) controls were used to determine positivity. PDGFRα+/CD56− fraction was defined as FAPs. FAPs were expanded *in vitro* for a total of 1 week until obtaining two confluent flasks to collect for cell suspension.

### 2.4 10X single cell RNA-seq

The cell suspension obtained from each sample was transferred to the Single Cell Genomics laboratory at the National Center for Genetics in Barcelona (CNAG) on ice in DMEM+5%FBS. Cells were centrifuged at 300 rcf during 5 min at 4° in order to bring the cell concentration to 300–1,000 cells/μL. Cell concentration and viability were determined by manual counting using a Neubauer chamber and staining the cells with Trypan blue. Cells were partitioned into Gel Bead-In-Emulsions (GEMs) by using the Chromium Controller system (10X Genomics), with a target recovery of 5,000 total cells per sample. cDNA sequencing libraries were prepared using the Next GEM Single Cell 3′ Reagent Kits v3.1 (10X Genomics, PN-1000268), following manufacturer’s instructions. Briefly, after GEM-RT clean up, cDNA was amplified during 12 cycles and cDNA QC and quantification were performed on an Agilent Bioanalyzer High Sensitivity chip (Agilent Technologies). cDNA libraries were indexed by PCR using the PN-220103 Chromiumi7 Sample Index Plate. Size distribution and concentration of 3′cDNA libraries were verified on an Agilent Bioanalyzer High Sensitivity chip (Agilent Technologies). Finally, sequencing of cDNA libraries was carried out on an Illumina NovaSeq 6,000 using the following sequencing conditions: 28 bp (Read 1) + 8 bp (i7 index) + 0 bp (i5 index) + 89 bp (Read 2), to obtain approximately 20–30.000 reads per cell.

### 2.5 Real time PCR

Total RNA was extracted from cell pellets using PureLink RNA Mini Kit (Invitrogen) and quantified using nanodrop ND-100 spectrophotometer (Nanodrop Technologies, Wilmington, DE, United States). Five hundred ng of total RNA was retrotranscribed using the High Capacity RT Kit (Applied Biosystems, Foster City, CA) following manufacturer’s instructions. qPCR was performed by triplicate using the Fast Advanced Master Mix and a 7900 HT Fast Real-Time PCR system (ThermoFisher). The mRNA-specific Taqman probes (Applied Biosystems) were used for the different genes. Relative quantification was performed using the comparative Ct method. GAPDH was used as endogenous control.

### 2.6 Immunofluorescence

Frozen muscle sections were obtained with a cryostat (Leica Microsystems, Wetzlar, Germany), fixed with acetone, and incubated with blocking solution (Santa Cruz Biotech). The antibodies used in the study are listed in Table 2.

**TABLE 2:** Information about the antibodies used in this study.

Target	Clone	References	Host
CHI3L1	YKL40	PA5-43746	Rabbit
EFEMP1	EPR22855-4	ab 256457	Rabbit
FGF7	KGF/FGF-7	ab 214178	Rabbit
PTX3	MNB-1	ab 90806	Rat
TGFβRII	EPR23237-2	ab 270440	Rabbit
MFAP5	EPCSUR1	ab 171737	Rabbit

### 2.7 Bioinformatic analysis

We used Cellranger mkfastq version 3.01 to de-multiplex the BCL files to FASTQ files. Using Cellranger count and the human reference genome GRCh38, we aligned the reads and quantified them. We checked the quality of the cells in each sample in R (version 4.2.1). For downstream analysis, cells with less than 1,000 reads or 500 genes and more than 5% mitochondrial reads were filtered out (Wolock et al., 2019). Using DoubletFinder (version 2.0.3), we identified and filtered out the doublets from each sample. The standard clustering workflow in Seurat (version 4.3.0) was used to analyse the samples (Stuart et al., 2019). We performed SCTransform (version 0.4.0) normalisation to remove cell to cell differences, and then scaled the data. 2000 highly variable genes were then identified using which we conducted principal component reduction and clustering of the data. Harmony (version 0.1.1) was used to integrate the samples. The data was then visualised using a Uniform Manifold Approximation and Projection (UMAP) plot and clusters were identified. We used FindMarkers function with Wilcoxon test method to identify the marker genes for each cluster. These markers along with those present in literature were used to identify the cell types represented by the clusters. Raw and processed sequencing data are available under request to the corresponding author.

### 2.8 Statistics

We used the Shapiro-Wilk test to confirm that none of our variables were normally distributed. As such, non-parametric statistic tests were used for the analysis. Statistical analyses and graphic representations were per-formed with GraphPad Prism Software 8 (La Jolla, CA, United States).

## 3 Results

### 3.1 Cell heterogeneity in healthy and DMD isolated FAPs

**FIGURE 1:**
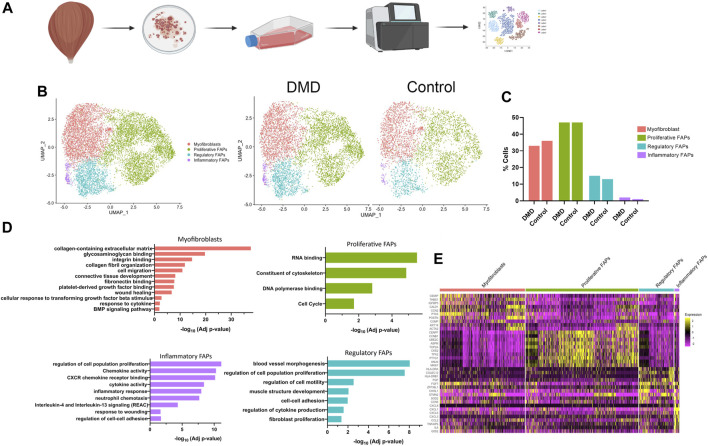
Classification of cell types in healthy and DMD FAPs. **(A)** Project design. Muscle explants were cultured for 6 days until obtaining surrounding cells. After sorting, FAPs were expanded for 1 week until obtaining two confluent flasks. **(B)** UMAP visualization of all the cells from healthy and DMD samples coloured by cluster identity. **(C)** Bar graph showing the percentage of cells identify in each cluster identity both in healthy FAPs and DMD FAPs. **(D)** Top biological processes upregulated in each cluster. **(E)** Heatmap showing expression of genes involved in each cluster.

We used scRNA-seq analysis to study the gene expression profile of FAPs arising from skeletal muscle explants of three patients with DMD and two age and gender matched controls to capture gene expression signatures that could identify different cells populations (Figure 1A). Sorted FAPs were expanded *in vitro* for 1 week before being collected for scRNAseq. We analysed a total of 10,000 cells across all the experimental conditions. A total of 2,000 cells per sample was analysed to normalize any potential difference and detected a total of 3,677 genes with a read depth between 30 and 50 k per sample. UMAP, used for dimensionality reduction, revealed no batch effect between samples from the different patients (Supplementary Figure S1) These analyses identified 4 clusters of cells, likely representing different stages of cell differentiation of FAPs that were present both in DMD and control samples (Figure 1B). We did not observe any cluster of cells only present in controls or DMD and we did not find differences in the proportion of cells belonging to each cluster between controls and DMD (Figure 1C). Cluster one was characterized by the expression of genes coding for components of the extracellular matrix, organization of collagen fibril, cell migration, and genes involved in binding to glycosaminoglycans, integrin or fibronectin. Based on their gene expression profile, we named this cluster as myofibroblasts. Cluster two, which was the predominant cluster, was characterized by the expression of genes involved in cell division, RNA binding and cell cycle and we named this cluster as proliferative cells. Cluster three was characterized by the expression of genes involved in regulation of cytokine production, cell to cell adhesion, regulation of cell motility and regulation of cell proliferation and we named this cluster as regulatory FAPs. Cluster four was characterized by the expression of genes involved in inflammatory response, cytokine and chemokine activity, *IL4* and *IL13* response and neutrophil chemotaxis and we named this cluster as inflammatory FAPs (Figure 1D). Figure 1E displays the heatmaps of the top ten upregulated genes by each defined cluster.

### 3.2 Transcriptional signature of clusters

**FIGURE 2:**
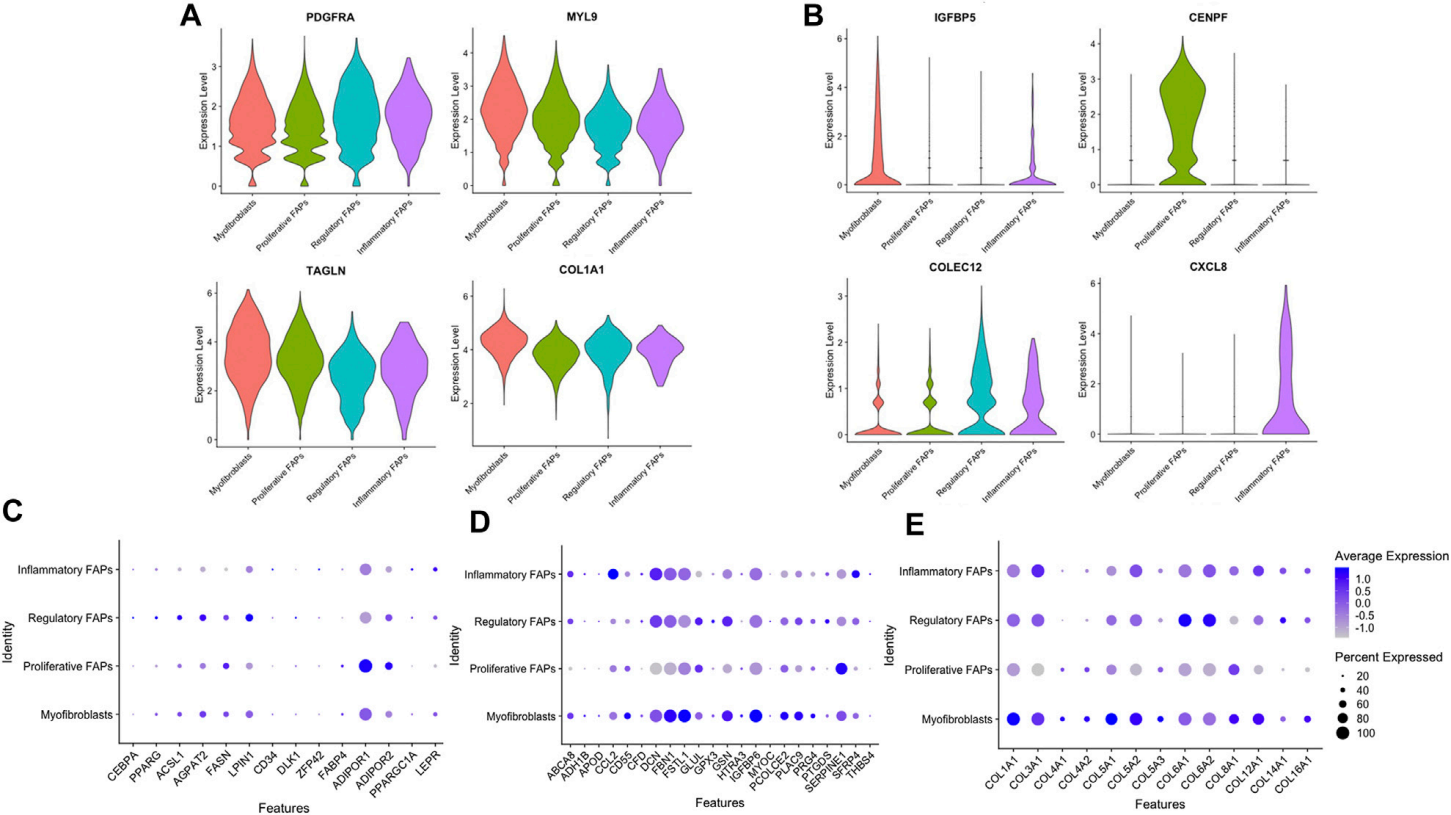
Expression levels of distinct genes in the different clusters. **(A)** Violin plot showing the expression levels of several genes heterogeneously expressed in the different clusters. **(B)** Violin plot showing the expression levels of distinct genes upregulated in each cluster. **(C)** Selected genes related to adipogenesis expressed in each FAP cluster. **(D)** Selected genes related to fibrogenesis expressed in each FAP cluster **(E)** Selected genes related to extracellular collagens expressed in each FAP cluster.

Most of the cells repositioned into a heterogenous population in which clusters were not clearly separated since some genes were equally expressed in all of the clusters, such as *PDGFRA, MYL9, TAGLN and COL1A1* (Figure 2A). However, we found several genes that were specifically upregulated in each of the different clusters. *IGTBP5* and *CEMIP* were highly expressed in myofibroblasts, *CENPF* expression was mainly expressed in proliferative FAPS, *HLA-DRA* and *COLEC12* were upregulated in regulatory FAPs and *CXCL8* was only expressed in inflammatory FAPs (Figure 2B).

Since FAPs are known for their capacity to differentiate into either adipocytes and fibroblasts *in vivo*, we assessed if there were differences in the expression of genes related to fibrogenesis, adipogenesis and extracellular collagens among the different cell clusters. We observed that the percentage of cells expressing these genes were similar among the different clusters, although some of these genes had a higher expression than others. Proliferative FAPs was the cluster with more differences in the expression levels of these genes compared with the other clusters. For example, when analysing the adipogenesis genes (Figure 2C), *ADIPOR1* was highly expressed in proliferative FAPS compared to myofibroblasts, regulatory and inflammatory FAPS while some of the fibrogenic genes (Figure 2D) such as *DCN, FBN1, IGBP6* or several genes encoding collagen (*COL3A1, COL5A2, COL6A1, COL6A2*) were also less expressed by proliferative FAPs than by the other clusters. (Figure 2E). Unfortunately, we could not identify any transcriptional signature that was completely specific of a cluster that could be used to isolate cells from muscle or cell culture using sorting. Based on these results, we speculate that FAPs share a core of genes that are expressed by all cells types regardless of their differentiation stage, as a genetic signature that could be characteristic of FAP cells. However, once FAPs adopt specific functions, they change the gene expression profile accordingly as we have observed in our study. We suggest that the clusters that we have identified are composed by cells in dynamic transition from one cluster to another, rather than representing cells that have irreversibly differentiated into a specific cell types.

### 3.3 DEG analysis reveals differential gene expression between healthy and DMD FAPs

**FIGURE 3:**
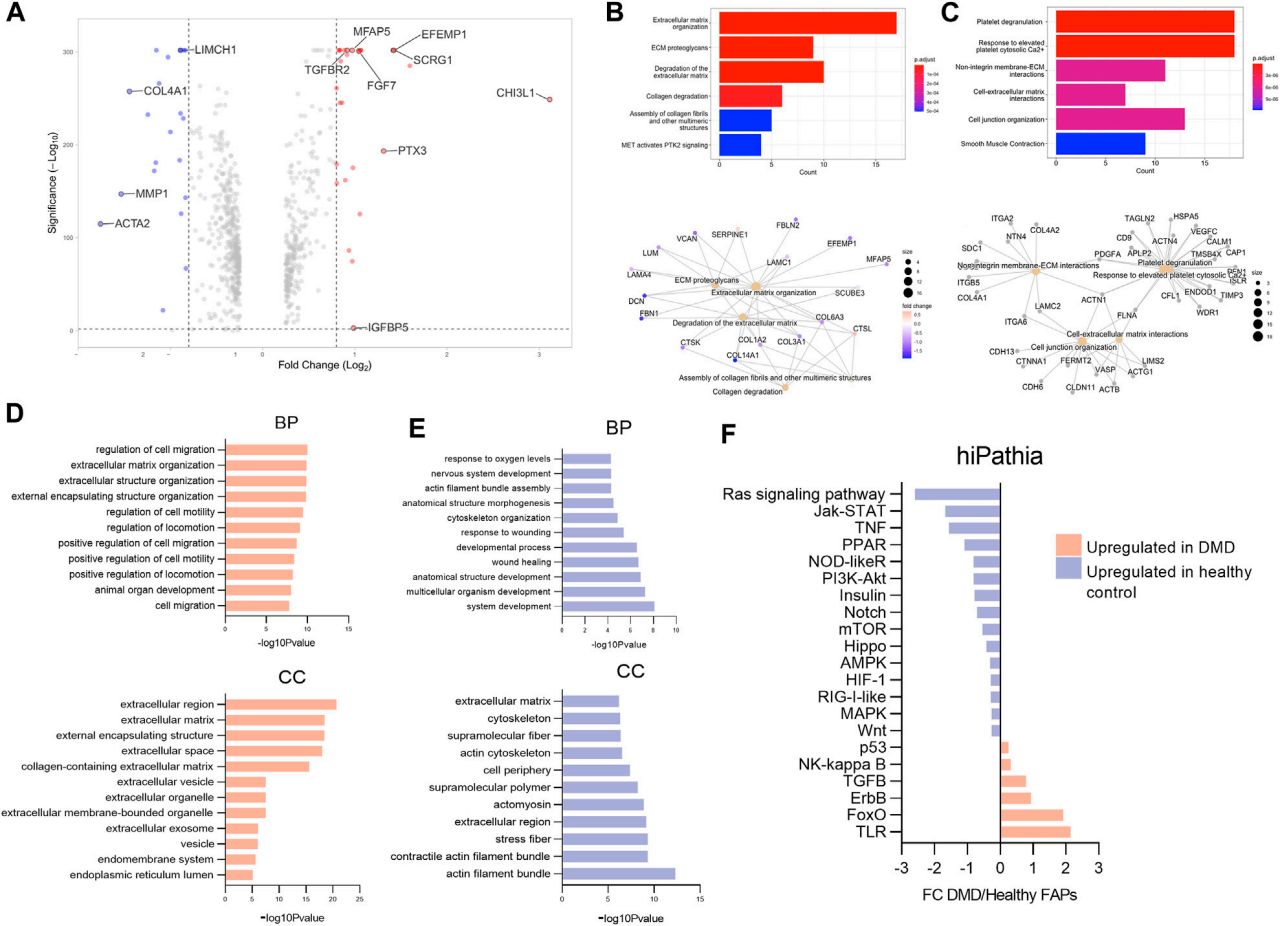
Analysis of gene expression changes in DMD FAPs compared to healthy control FAPs. **(A)** Volcano plot representing Fold change (log2) and *p*-value (-log10) from DMD FAPs genes compared to healthy FAPs. **(B)** Selected gene ontology (GO) terms and interaction network analysis showing the functional relationships among specific genes in DMD FAPs. **(C)** Selected gene ontology (GO) terms and interaction network analysis showing the functional relationships among specific genes in healthy FAPs. **(D)** Results of the enriched terms along with the statistical significance (-log10 *p*-value) according to the gene ontology biological process (BP) and the gene cellular compartment (CC) upregulated in DMD. **(E)** Results of the enriched terms along with the statistical significance (-log10 *p*-value) according to the gene biological process (BP) and the gene cellular compartment (CC) upregulated in healthy control FAPs. **(F)** Results of the enriched terms along with the statistical significance (-log10 *p*-value) according to the gene cellular compartment (CC) upregulated in healthy control FAPs. Barplot representing the molecular pathways upregulated in DMD FAPs or in healthy FAPs using hipathia program (http://hipathia.babelomics.org).

We identified 2056 genes that were differentially expressed between healthy control and DMD FAPs, as illustrated in the volcano plot on Figure 3A. The most upregulated genes in DMD based on fold change were *CHI3L1*, *EFEMP1, MFAP5, TGFBR2, SCRG1, FGF7, IGFBP5* and *PTX3* (FDR corrected *p*-value<0.05). Conversely, in healthy FAPs, the most highly expressed genes were *ACTA2, MMP1, COL4A1* or *LIMCH1*. Supplementary Table S1 show a list of the top genes that were upregulated in control and DMD samples.

In order to determine the physiological roles of the identified differentially expressed genes, we performed Gene Ontology enrichment analysis using Reactome database. Our analysis indicates that the molecular functions enriched in DMD FAPs were related to ECM organization, ECM degradation and collagen degradation (Figure 3B). We performed an interaction network analysis to identify the functional relationships among the specific genes upregulated in DMD FAPs and the molecular functions analysed using the STRING database. Our analysis revealed that these genes formed a significant functional network involved in different extracellular matrix organization functions (Figure 3B). When analysing the enriched genes in healthy FAPs, we observed molecular functions related to platelet degranulation, non-integrin membrane ECM interaction and cell-extracellular matrix interaction (Figure 3C). To further characterize the gene differences between DMD and health FAPs, we analysed a list of differentially expressed genes in terms of enriched gene ontology categories for upregulated and downregulated genes. The results obtained from the analysis of upregulated genes using the g:Profiler indicated major categories of gene expression enrichment according to the gene ontology molecular biological process (GO-BP) or cellular compartment (GO-CC). Regarding the biological processes, the most enriched categories identified in DMD FAPs were those related to cell migration and extracellular matrix organization, which are biological processes in agreement with the cellular compartment categories identified in this study, as most of them were related to extracellular region (Figure 3D). Conversely, when we analysed the biological processes upregulated in healthy FAPs, the most enriched categories were those related to system development, wound healing and cytoskeleton organization and the most enriched cellular compartment categories were actin filament and cytoskeleton compartment (Figure 3E). Taken together, our data suggest that genes upregulated in DMD FAPS are involved in extracellular matrix dynamics and cell migration while genes upregulated in healthy FAPs are involved in structural and cytoskeleton organization.

To obtain a complete picture of the variations in gene expression between DMD and control FAPs we analyzed potential changes in signalling pathways using high throughput pathway interpretation and analysis (hiPathia) tool, which transforms uninformative gene expression data into signalling pathway circuit activities (Hidalgo et al., 2016; Rian et al., 2021) (Figure 3F). The increased cell signalling circuits identified allowed us to estimate the signalling activity profiles obtained from the transcriptomic data. As shown in Figure 3F, signalling circuits related to Toll-like receptor (TLR), p53, foxO, Nf-kB, erbB and TGFβ were upregulated in the DMD FAPs condition while circuits related to Ras signalling, Kat-STAT, TNF or Notch pathways were upregulated in the healthy control FAPs condition.

### 3.4 Validation of the identified enriched genes in human tissue

**FIGURE 4:**
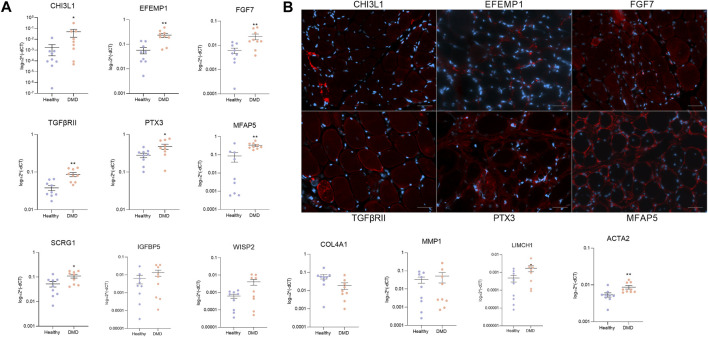
Validation of specific genes. **(A)** qPCR analysis of specific upregulated genes in FAPs from DMD patients (*n* = 8) and FAPs from aged-matched healthy controls (*n* = 9). **(B)** Representative immunofluorescence images of CHI3L1, EFEMP1, FGF7, TGFβRII, PTX3 and MFAP5 in DMD muscle biopsy. Scale bar = 50 µM. Data are represented as the mean of three replicates ±standard error of the mean. Statistical significance was set at *p* < 0.05. **p < 0.01; ***p < 0.001.

We validated the results obtained in the scRNAseq either in cells cultured *in vitro* and also in muscle tissue obtained from a DMD patients and healthy aged matched controls. First, we studied the expression of a subset of the enriched genes in both DMD and healthy FAPs by qPCR using FAPs isolated from eight different DMD patients and nine aged-matched healthy controls. Our analysis showed that *Chi3l1*, *Efemp1*, *Fgf7*, *TgfbrII*, *Ptx3*, *Mfap5*, *Scrg1* and *Igfbp5* genes were expressed at higher levels in DMD FAPs *in vitro*, confirming the results observed with scRNA-seq analyses (Figure 4A).

We also assessed the protein expression of the identified enriched genes in muscle samples of DMD patients using immunofluorescence. We observed that the most enriched genes obtained by the scRNAseq analysis were translated and expressed in human tissue as it is shown in Figure 4. We also analysed the co-localization of the selected genes with FAPs in muscle tissue from healthy aged matched control and DMD patient. We observed that CHI3L1, EFEMP1, FGF7, TGFBRII, PTX3 and MFAP5 were more present in the DMD condition compared to the healthy condition (Supplementary Figure S2). These data validate the results observed in our scRNAseq studies of cultured FAPs and confirm that the genes identified *in vitro* can also be found *in vivo* in human muscle tissue, opening the door to study pathophysiology of these diseases (Figure 4B).

## 4 Discussion

In this study, we profiled the transcriptome of cultured FAPs isolated from human skeletal muscle to investigate the existence of subtypes of FAPs and their gene signature. We have studied FAPs isolated from DMD patients and age and gender matched controls and have identified transcriptional differences among both groups. Overall, our results suggests that there are significant differences in the gene expression profile of DMD FAPs compared to controls, suggesting that DMD FAPs are actively participating in the reorganization and expansion of the extracellular matrix that takes place during the process of muscle degeneration in DMD.

It is well known that FAPs contribute to maintain tissue homeostasis and support muscle regeneration in healthy muscle (Farup et al., 2015). After an acute muscle damage, FAPs proliferate and release components of the extracellular matrix that serve as scaffolds for the newly formed muscle fibres, but FAPs also secrete cytokines and growth factors that stimulate satellite cell activation and proliferation (Tedesco et al., 2010; Murphy et al., 2011). However, in muscular dystrophies, FAPs can adopt different behaviour characterized by continuous proliferation and enhanced expression of extracellular matrix components, such as collagens, leading to fibrosis or differentiation into adipogenic cells contributing to the expansion of fat tissue usually observed in muscle samples of patients (Joe et al., 2010; Lemos et al., 2015). Moreover, it has been suggested that FAPs can also have a role in self-regulating their differentiation or perpetuating the infiltration of macrophages in the injured muscles. In front of this myriad of potential cell functions, it is a question of debate if there are subtypes of FAPs with a specific role in the process of degeneration. In this study, we aimed to analyse if there were differences in the gene expression profile of DMD FAPs isolated and grown *in vitro* compared to FAPs from healthy controls. We firstly studied if there were different subpopulations of FAPs present in the cells arising from muscle explants grown in culture and identified four major clusters of FAPs that could potentially have different functions: myofibroblasts, proliferative FAPs, regulatory FAPs and inflammatory FAPs. Interestingly, these different clusters shared the expression of several genes, included the ones that are classically used to distinguish FAPs from other cells, such as *PDGFRA, TAGLN* or several collagens such as *COL1A1* or *COLA3*, suggesting that FAPs share a core of genes regardless of their differentiation stage as a kind of genetic signature that could be useful to differentiate them from other muscle resident cells. Although we observed differences in the gene expression profile between clusters, we were not able to identify a group of genes that was only expressed in one cluster and could be used for isolating it either from fresh muscle or from the mixed population *in vitro* for further experimentation.

As clusters shared the expression of several genes, we speculate that the population of FAPs studied *in vitro* is composed by cells in dynamic transition from one cluster to another, rather than by different cell types. In fact, we observed a similar pattern of gene expression between clusters when analysing specific groups of genes such as those involved in fibrogenesis (including several collagens) and adipogenesis. Previous studies have investigated FAP heterogeneity at a single level in humans by evaluating the cellular composition of FAP population in muscle from healthy volunteers (De Micheli et al., 2020). Rubenstein et al. identified two distinct subpopulation of FAPS that were characterized by the expression of *FBN1* and *LUM* genes (Rubenstein et al., 2020). More recently, Fitzgerald et al. used single cell RNA seq and single-nuclei to characterize FAP heterogeneity in patients with fatty infiltration. They observed an *MME +* FAP subpopulation that exhibited high adipogenic potential (Fitzgerald et al., 2023). We did not observe specific clusters enriched in adipogenic genes, however, this could be due to technical reasons, as adipogenic cells are easily lost during the processing steps for single cell sequencing, or to the fact that profibrotic cells are majority in the skeletal muscles of patients with DMD.

Conversely, we identified differences in the expression profile of DMD FAPs compared to control cells. We validated these differences in gene expression *in vitro* by qPCR in a larger number of cell samples and further confirmed its expression at the protein level in muscle tissue obtained from DMD patients, suggesting that the specific pathological settings observed in scRNAseq results are translated at the protein level in muscle tissue.

In order to understand the biological contribution of the differentially expressed genes in DMD FAPs we used several tools to profile the biological pathways activated in these cells. DMD FAPs predominantly activated the expression of genes involved in extracellular matrix organization and cell migration while healthy FAPs predominantly activated genes related to cytoskeleton organization and actin filament assembly. These results are in agreement with data recently published by our group, where we have used snRNAseq to study muscle samples of patients with DMD and healthy controls. In that study, we observed that DMD FAPs were characterized by the expression of several genes involved in the production and remodelling of extracellular matrix, but also genes involved in DNA replication and cell proliferation as we have observed in the present study (Suárez-Calvet et al., 2023). These results were confirmed *in vivo* in patients with DMD and Becker muscular dystrophy, where we observed an increased number of FAPs associated to an expanded amount of extracellular matrix mainly composed of collagen I and VI (Pinol-Jurado et al. under review).

When studying differences in the signalling pathways gene profile, we observed that TLR, FoxO, ErbB or TGF-β were the most increased pathways in DMD FAPs while Ras, Jak-STAT, TNF or PPARγ were more relevant in healthy FAPs. Some of these results are in agreement with previous studies in FAPs. For example, TGF-β has been studied as a key mediator of fibrogenesis in muscle dystrophies and not surprisingly, it was also enhanced in DMD FAPs (Vetrone et al., 2009; Nelson et al., 2011; Mázala et al., 2020)^.^ TLR signalling was the highest increased pathway in DMD FAPs in our study. Although its role on FAPs has not been addressed yet, previous studies using muscle samples of patients with limb girdle muscular dystrophy R2 (dysferlin-mutated), have shown that AnxA2 activates TLR pathway after muscle injury promoting FAP proliferation and adipogenesis. Additionally, inhibiting TLR signalling reduced muscle pathology in murine models of dysferlinopathy (Uaesoontrachoon et al., 2013). Since our study showed that the TLR pathway is increased in DMD FAPs, it is tempting to hypothesize that there is a potential role for TLR pathway in FAPs expansion and differentiation. Other mechanisms identified in our study are the Forkhead box O (FOXO) family or the ErbB signalling pathway. Neither of these mechanisms have been characterized in FAPs yet, however their role on cancer metastasis and in the epithelial-to-mesenchymal transition in cancer associated fibroblasts suggests that a similar role could be observed in FAPs from muscle dystrophies. Overall, the molecular pathways increased in the DMD FAPs are in accordance with the biological processes observed in the enrichment analysis, since these pathways are related with ECM reorganization and FAP migration. Regarding the pathways increased in healthy FAPs, some of them have already been studied in the homeostasis role of FAPs in muscle regeneration. Healthy FAPs contribute to tissue regeneration by supporting differentiation of SCs and by reabsorbing the excess of ECM (Santini et al., 2020). We observed an increased expression of genes belonging to Jak-STAT, TNF-α, Notch or Wnt pathways which have been described to promote a pro-regenerative role of FAPs rather than influencing their fibrogenic and adipogenic potential (Lemos et al., 2015; Kopinke et al., 2017; Reggio et al., 2020). For example, Jak-STAT pathway activated by IL-15 has been suggested to stimulate FAP proliferation and prevent of FAP differentiation and fatty degeneration after muscle damage (Kang et al., 2018). TNF-α signaling promotes FAP apoptosis after acute muscle damage, limiting the continuous expansion of these cells and avoiding fibrotic deposition in the injured muscle while Notch and Wnt suppress adipogenesis (Ross et al., 2000). The pro-regenerative role of these pathways together with anti-fibrogenic and adipogenic differentiation function, is consistent with the biological processes observed in our analysis. We observed that the structural and actin organization molecular pathways were increased in FAPs obtained from healthy controls which could be relevant in the maintenance of FAP homeostasis, as this could promote muscle regeneration. Thus, it is not surprising that decrease in such signaling pathways are often found in human muscle wasting diseases that are complicated by FAP-derived adipose and fibrous infiltrates such as DMD.

scRNA-seq has emerged as a powerful tool to profile the transcriptome of thousands of individual cells in one single experiment (Stuart and Satija, 2019). ScRNA-seq analysis permits an unbiased survey of cellular complexity and heterogeneity involving the regulation occurring at the transcriptional level. Although scRNAseq results need to be further validated in larger number of cells or tissues using protein analysis, scRNAseq provides a deep insight into the different genetic signatures of cells from DMD and controls. In our study, we provided for the first time data on scRNAseq from FAPs isolated from DMD and age and gender healthy matched controls isolated from skeletal muscles and cultured *in vitro.* One of the limitations of our study is the number of samples used and the expansion of FAPs *in vitro* before performing the scRNAseq as this is not fully representative of the *in-vivo* condition. Due to the low availability of DMD samples, we wanted to maximise the low amount of tissue obtained to perform a transcriptomic study. While scRNAseq from muscle tissue requires at least 50–65 mg of muscle (De Micheli et al., 2020.), here we used less than 2 mg of muscle. However, we had to expand sorted cells *in vitro*, something that can interfere with the *in vivo* transcriptome profile. Although *in vitro* studies cannot completely mimic what occurs *in vivo*, we validated the top genes found in muscle tissue from DMD and healthy patients and corroborated that the increased genes that we observed in our analysis were maintained in the DMD muscle compared to the healthy muscle.

Taken together, we have identified FAPs differential expression between healthy controls and DMD patients. Understanding why DMD FAPs are different and which signals promote this difference may provide potential targets for new therapeutical approaches to prevent muscle degeneration. We believe that using the single-cell RNA seq from human samples may provide new opportunities to study cellular coordination to further understand the regulation of muscle homeostasis and degeneration that occurs in muscular dystrophies.

## 5 Scope statement

Our manuscript “Single cell RNA sequencing of human FAPs reveals different functional stages in Duchenne muscular dystrophy” submitted to Frontiers in Cell and Developmental Biology. Our work uses primary cells and single-cell transcriptomics to analyse the single cell transcriptome of fibroadipogenic precursor cells (FAPs) obtained from healthy and DMD muscle tissue. Our scRNA-seq experiments revealed that there are specific de-regulated genes between the different conditions that are in accordance with different biological function. The most significant genes have been transcriptionally validated in a larger number of FAP cells from both DMD patients and healthy age-matched controls and histologically validated in DMD muscle tissue. This is the first work that identifies gene expression using primary cells isolated from DMD patients and healthy aged-matched controls and studies the molecular mechanisms differentially expressed in pathogenic conditions.

## Data Availability

The data presented in the study are deposited in the Single cell Broadinstitute.org repository, accession number SCP2678, available at: https://singlecell.broadinstitute.org/single_cell/study/SCP2678/single-cell-rna-sequencing-of-human-faps.
