# Supplementary material for: Single cell RNA sequencing of human FAPs reveals different functional stages in Duchenne muscular dystrophy
Source: Front Cell Dev Biol. 2024 Jul 9;12:1399319. doi: 10.3389/fcell.2024.1399319 (PMC11264872; doi:10.3389/fcell.2024.1399319)
Supplement: Supplementary file 1 [file Table1.DOCX]

| **Gene** | **p_val** | **avg_logFC** | **p_val_adj** |
| --- | --- | --- | --- |
| CHI3L1 | 1.28E-249 | 3.112961 | 2.28E-245 |
| CCN5 | 6.77E-286 | 1.597462 | 1.20E-281 |
| SCRG1 | 0 | 1.421003 | 0 |
| EFEMP1 | 0 | 1.416853 | 0 |
| PTX3 | 3.39E-194 | 1.312698 | 6.03E-190 |
| SERPINF1 | 0 | 1.06962 | 0 |
| MGST1 | 0 | 1.062072 | 0 |
| PTGDS | 2.76E-126 | 1.054996 | 4.91E-122 |
| FGF7 | 1.38E-301 | 1.046186 | 2.46E-297 |
| C1S | 0 | 1.039494 | 0 |
| IGFBP5 | 0.001121 | 0.985807 | 1 |
| G0S2 | 3.84E-176 | 0.979689 | 6.82E-172 |
| SOD2 | 1.66E-75 | 0.97255 | 2.96E-71 |
| MFAP5 | 0 | 0.971573 | 0 |
| TNFAIP6 | 5.13E-87 | 0.935841 | 9.12E-83 |
| TGFBR2 | 0 | 0.916991 | 0 |
| PFN1 | 0 | -0.85537 | 0 |
| TFPI | 3.13E-229 | -0.8595 | 5.56E-225 |
| KRT18 | 1.24E-126 | -0.88295 | 2.21E-122 |
| INHBA | 1.12E-234 | -0.88956 | 1.99E-230 |
| LIMCH1 | 0 | -0.89096 | 0 |
| FLNA | 0 | -0.89114 | 0 |
| MALL | 2.86E-184 | -0.89924 | 5.09E-180 |
| TIMP3 | 0.15858 | -0.97954 | 1 |
| RPS2 | 1.23E-214 | -0.99916 | 2.19E-210 |
| ANK3 | 3.10E-295 | -1.0255 | 5.50E-291 |
| SFRP1 | 1.10E-22 | -1.08033 | 1.96E-18 |
| COL4A2 | 7.43E-267 | -1.12179 | 1.32E-262 |
| NEFM | 0 | -1.15161 | 0 |
| PLAT | 1.24E-181 | -1.15806 | 2.20E-177 |
| CLDN11 | 1.02E-172 | -1.17493 | 1.81E-168 |
| TFPI2 | 2.64E-233 | -1.24393 | 4.69E-229 |
| COL4A1 | 2.71E-258 | -1.44372 | 4.82E-254 |
| MMP1 | 7.73E-148 | -1.53352 | 1.37E-143 |
| ACTA2 | 1.13E-115 | -1.75795 | 2.00E-111 |

**Supplemental table 1.** Top genes upregulated and downregulated in healthy and DMD FAPs.
