# Supplementary figures and images for: Single cell RNA sequencing of human FAPs reveals different functional stages in Duchenne muscular dystrophy
Source: Front Cell Dev Biol. 2024 Jul 9;12:1399319. doi: 10.3389/fcell.2024.1399319 (PMC11264872; doi:10.3389/fcell.2024.1399319)

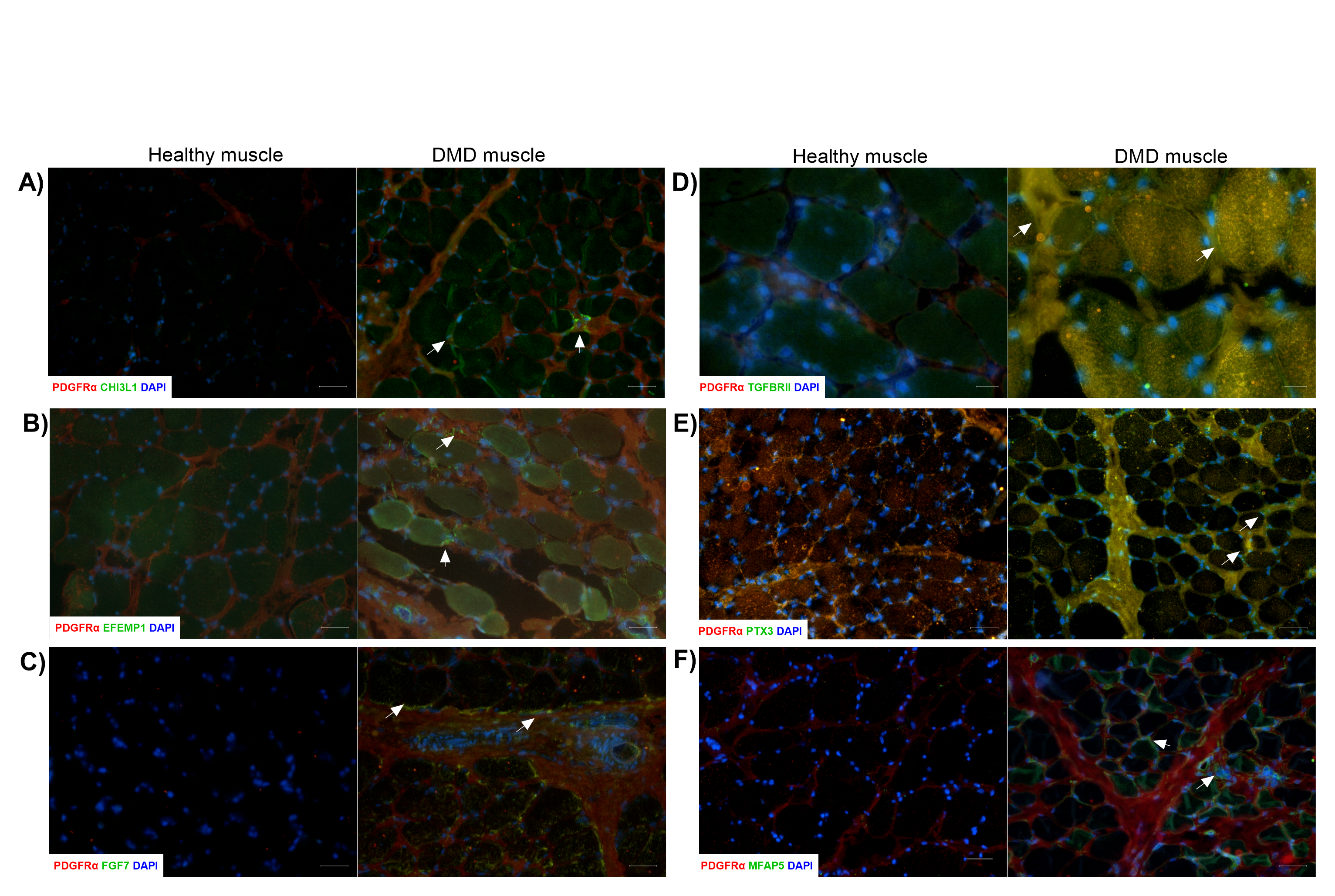

Supplement: Supplementary file 2 [file Image2.TIF]

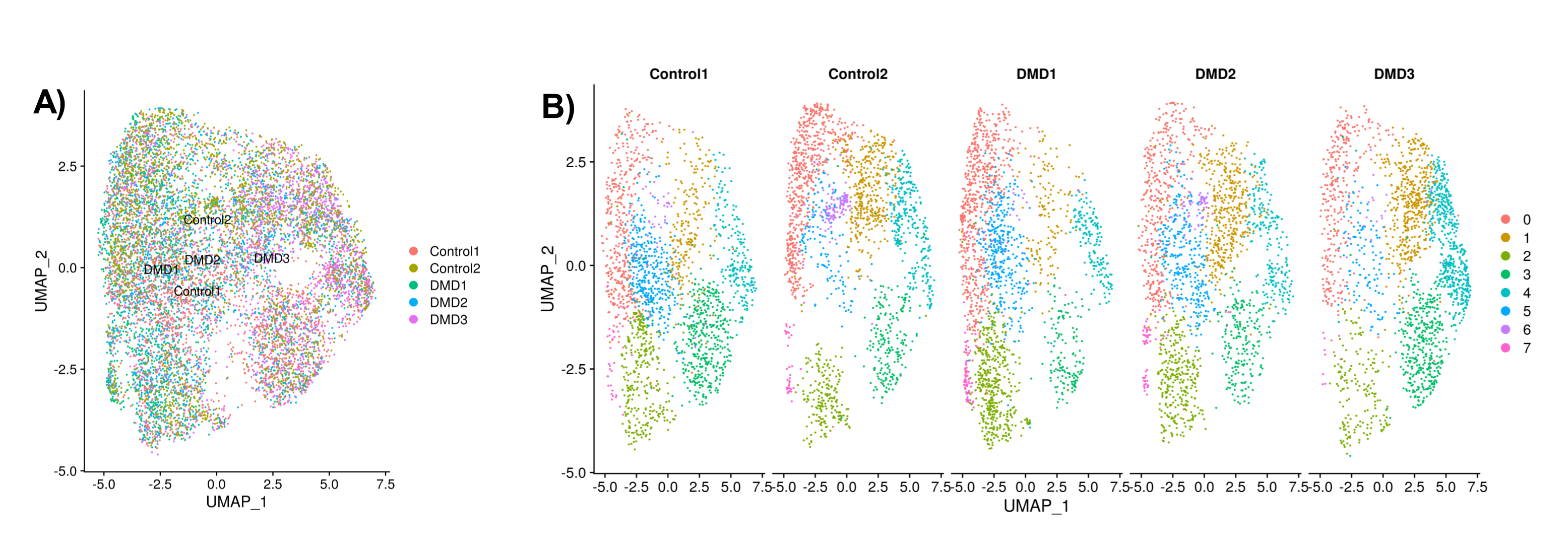

Supplement: Supplementary file 3 [file Image1.TIF]
